# Chemokine System Changes Drive Age-Related Macular Degeneration and Influence Treatment Outcomes

**DOI:** 10.1167/iovs.66.5.14

**Published:** 2025-05-06

**Authors:** Alexander Kai Thomsen, Maria Abildgaard Steffensen, Amalie Thomsen Nielsen, Henrik Vorum, Bent Honoré, Mogens Holst Nissen, Torben Lykke Sørensen

**Affiliations:** 1 Department of Ophthalmology, Zealand University Hospital, Roskilde, Denmark; 2 Department of Clinical Medicine, University of Copenhagen, Copenhagen, Denmark; 3 Department of Immunology and Microbiology, University of Copenhagen, Copenhagen, Denmark; 4 Department of Clinical Medicine, Aalborg University, Aalborg, Denmark; 5 Department of Ophthalmology, Aalborg University Hospital, Aalborg, Denmark; 6 Department of Biomedicine, Aarhus University, Aarhus, Denmark

**Keywords:** chemokine, chemokine receptor, age-related macular degeneration (AMD), neovascular AMD (nAMD), treatment response

## Abstract

**Purpose:**

The chemokine system is associated with age-related macular degeneration (AMD), shown in previous studies. In this study, we investigate these chemokines and chemokine receptors and their association with treatment response in neovascular AMD (nAMD), and association to intermediate AMD (iAMD).

**Methods:**

In this prospective cohort study, patients with nAMD, iAMD, and healthy controls were included. The initial and 1-year treatment response was evaluated in patients with nAMD. Plasma chemokine concentrations of CCL2, CCL3, CCL4, CCL20, CXCL8, and CXCL10 were measured with immunoassays. Chemokine receptor expression levels of CCR1, CCR2, CCR5, CCR6, CXCR2, CXCR3, and CX_3_CR1 on circulating T cells and monocytes were measured with flow cytometry. Correlation network analyses were performed of the chemokine system. Genotyping for CFH and ARMS2 risk polymorphisms was performed in patients with nAMD.

**Results:**

Patients with nAMD with poor initial treatment response had significantly lower proportions of CD4+CXCR3+, CCR5+ classical monocytes, and CCR2+ non-classical monocytes compared with good initial responders (all *P* < 0.05). Patients with nAMD with poor 1-year treatment response had significantly lower CD4+CXCR3+ and CCR2+ non-classical monocytes compared to good 1-year responders (both *P* < 0.05). Correlation networks revealed a more complex regulation in partial and poor initial treatment responders. Multiple chemokines and chemokine receptors significantly correlated with the risk genotypes of CFH and ARMS2.

**Conclusions:**

Patients with nAMD with poor treatment response had dysregulation of the chemokine system. The chemokine system might be a potential target of novel treatment in nAMD. Further studies are needed to clarify the chemokine system’s role in nAMD treatment response.

Age-related macular degeneration (AMD) is a multifactorial disease and a common cause of visual impairment and blindness in the elderly, yet the treatment options are limited.^1^ In the late stage of neovascular AMD (nAMD), characterized by the formation of macular neovascularization (MNV), the pro-angiogenic vascular endothelial growth factor (VEGF) plays a key role. Treatment consists of repeated intravitreal anti-VEGF injections, which have improved visual outcomes for this patient group greatly. However, treatment response differs significantly between patients, and a considerable number will respond poorly with loss of vision despite the treatment.^2^ Novel treatment for the patients poorly responding to anti-VEGF injections are needed to halt the deterioration of vision and life quality of this patient group.^3^ No treatment options exist for patients with intermediate AMD (iAMD).^4^

Chronic low-grade inflammation is a key factor in the pathogenesis of AMD.^5^^,^^6^ Immunosenescence, a pro-inflammatory shift in the immune profile with aging, contributes to the angiogenic changes present in patients with AMD.^7^ A key component of the immune system is the chemokine system. Chemokines are cytokines that primarily induce chemotaxis, guiding leukocytes to sites of inflammation. Leukocytes, such as T cells and monocytes, have surface receptors for chemokines and will react upon stimulation by migrating to these sites and initiate their inflammatory and angiogenic response.^8^ The chemokine system is also affected by aging with increased systemic concentrations of pro-angiogenic chemokines,^9^ as well as alterations in expression levels of chemokine receptors on T cells^10^ and monocytes.^11^ Our group and others have previously found that systemic chemokines are associated with AMD, including C-C motif chemokine ligand 2 (CCL2), CCL3, CCL4,^12^^–^^14^ as well as chemokine receptors C-C motif chemokine receptor type 1 (CCR1),^15^ CCR2,^15^^,^^16^ CCR5,^17^^–^^19^ CCR6,^18^ C-X-C motif chemokine receptor type 3 (CXCR3),^18^^–^^21^ and C-X_3_-C motif chemokine receptor type 1 (CX_3_CR1).^16^^,^^17^^,^^22^ These chemokines and receptors regulate inflammation, which might contribute to the increased systemic inflammation observed in AMD.^5^ Bjerregaard and colleagues also found significantly increased levels of CD14+CCR1+ monocytes and decreased level of CD8+CCR3+ T cells in circulation between good and poor treatment responders in patients with nAMD.^23^

Genetic predisposition is also associated with AMD development. Single nucleotide polymorphisms of the complement factor H (CFH) and age-related maculopathy susceptibility 2 (*ARMS2*) genes have been shown to be associated with the stage of the disease.^24^^–^^26^ It has also been suggested that these polymorphisms are associated with treatment response,^27^ although this remains a subject of debate.^28^

Based on these prior findings, we investigated the relationship between the chemokine system and AMD stage (healthy, iAMD, and nAMD), as well as treatment response in patients with nAMD. Furthermore, the association between chemokines and chemokine receptors on mononuclear leukocytes and the risk genotypes CFH rs1061170 and ARMS2 rs10490924 in patients with nAMD was evaluated. These findings will contribute to the understanding of the chemokine system in patients with iAMD and patients with nAMD. Novel treatment targets might be identified in patients with nAMD with partial and poor treatment response to anti-VEGF injections.

## Methods

### Study Design and Participants

The Danish Neovascular Age-Related Macular Degeneration and Treatment Response (DANEART) study is a prospective cohort study. Systemic inflammatory and angiogenic biomarkers are analyzed and compared between AMD stages, as well as treatment response groups of patients with nAMD. The study was conducted at the Department of Ophthalmology, Zealand University Hospital, Denmark, approved by the Regional Committee of Ethics in Research of the Region of Zealand, Denmark (journal no: SJ-768), and performed in adherence with the Declaration of Helsinki. Verbal and written informed consent were obtained from all participants prior to inclusion.

Details on study design of the DANEART study has previously been reported,^6^ and are summarized below. Healthy controls, patients with iAMD, and patients with treatment-naïve nAMD were consecutively enrolled. Exclusion criteria were age younger than 60 years, active infections and cancer, inflammatory and autoimmune diseases, use of immunomodulating treatment, active smoking, plasma C-reactive protein (CRP) > 15 mg/L, previous anti-VEGF treatment, and other vision-affecting diseases than iAMD and nAMD.

Participants were examined at baseline, and patients with nAMD were additionally examined at two follow-up visits. To evaluate the initial treatment response, patients with nAMD were examined after a loading dose of 3 intravitreal anti-VEGF injections with monthly intervals, and the maintained treatment response after 1 year. Patients were treated according to Danish national guidelines with aflibercept, without switching, starting with a loading dose followed by individualized planning based on the observe-and-plan regimen.^2^ Participants were examined by retinal specialists in the clinic for best-corrected visual acuity (BCVA), slit-lamp biomicroscopy, color fundus photography, spectral domain optical coherence tomography (OCT) and OCT angiography. Classification of AMD stage was evaluated according to the Beckman criteria,^29^ and treatment response in patients with nAMD was graded according to persistence of intra- and subretinal fluid, and central retinal thickness on OCT. Good responders had total regression of retinal fluid, partial responders had persistence of fluid and decreased central retinal thickness (CRT), and poor responders had persistence of fluid and unchanged or increased CRT (Table 1).^6^^,^^30^

**Table 1.** Definitions of Treatment Responses to Intravitreal Anti-VEGF Injections

	Treatment Response
Good	Total regression of IRF and SRF
Partial	Persistence of IRF and/or SRF and reduction of CRT
Poor	Persistence of IRF and/or SRF and unchanged or increased CRT

CRT, central retinal thickness; IRF, intraretinal fluid; SRF, subretinal fluid.

Participants were examined for plasma chemokine concentrations and their receptor's expression level on T cells and monocytes (Table 2). Genotyping was also performed on patients with nAMD.

**Table 2.** Investigated Chemokines and Chemokine Receptor Pairs

Chemokine	Chemokine Receptor
CCL3	CCR1, CCR5
CCL4	CCR5
CCL20	CCR6
CCL2	CCR2
CXCL8	CXCR2
CXCL10	CXCR3
^*^	CX_3_CR1

CC, C-C motif; CXC, C-X-C motif; CX_3_C, C-X_3_-C motif; L, ligand’; R, receptor.

* The ligand of CX_3_CR1 is CX_3_CL1, which was not analyzed in this study.

### Chemokine Assays

All participants had blood sampled from the antecubital vein in lithium-heparin coated tubes and ethylenediamine tetraacetic acid (EDTA) coated tubes. Plasma chemokine concentrations were measured with immunoassays from the lithium-heparin coated tubes. These were first centrifuged at 1500 G for 15 minutes immediately after phlebotomy. The supernatant was isolated and frozen at –80°C within 1 hour to be analyzed on a different day. Chemokine assays were performed with electrochemiluminescence immunoassay V-plex analyzing for CCL2, CCL3, CCL4, CXCL8, and CXCL10, and V-plex analyzing for CCL20 (Mesoscale Discovery; see details in Supplementary Table S1). Duplicate analyses were performed according to the manufacturer’s guidelines.

### Flow Cytometry

**Figure 1.**
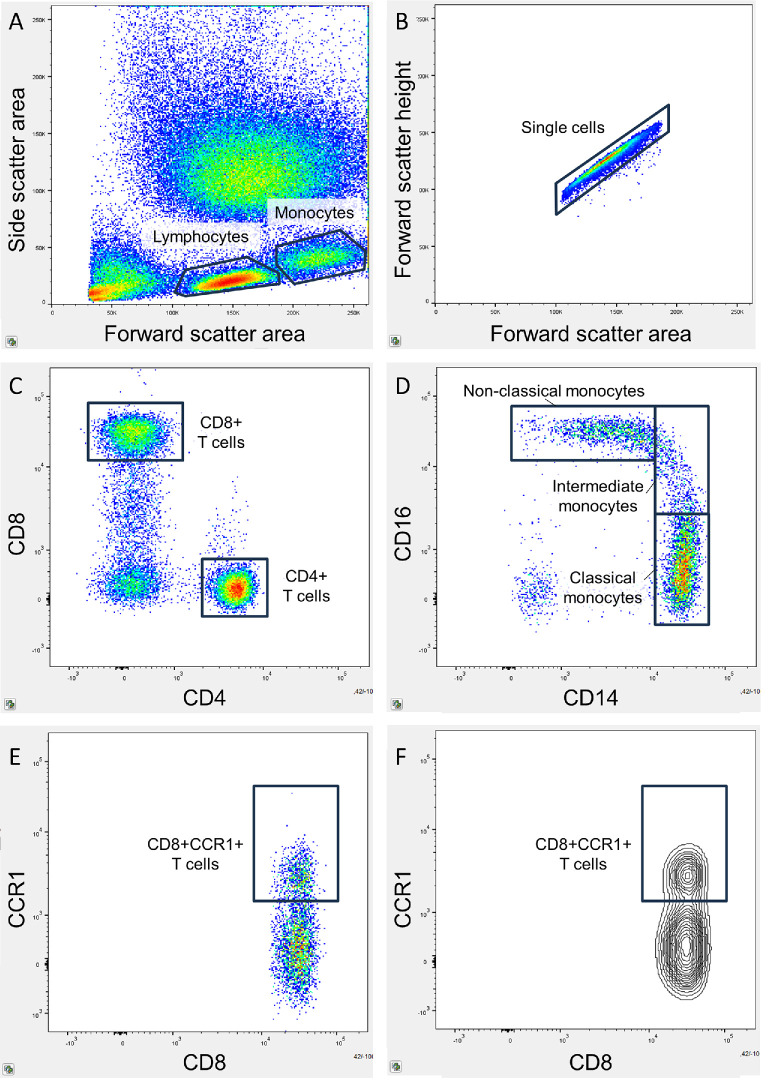
Flow cytometry gating strategy. (**A**) Lymphocytes and monocytes were identified on the forward-side-scatter plot. (**B**) Singlet cells were identified on the forward area-forward height plot. (**C**) Lymphocytes were gated for CD4 and CD8 to identify CD4+ and CD8+ T cells. (**D**) Monocytes were gated for CD14 and CD16, and monocyte subsets were identified as classical (CD14^high^CD16^low^), intermediate (CD14^high^CD16^high^), and non-classical (CD14^low^CD16^high^). (**E**) Chemokine receptors were gated, in this example CCR1 on CD8+ T cells using the pseudocolor plot and (**F**) contour plot.

Chemokine receptors were identified on T cells and monocytes with flow cytometry. The EDTA coated tubes were used for flow cytometry, which was initiated within 4 hours after phlebotomy. To determine the necessary blood volume for obtaining 1.0 × 10^6^ leukocytes for analysis, a leukocyte count on full blood was performed on the Sysmex KX-21NTM (Sysmex Corporation, Kobe, Japan). The determined volume was isolated, and erythrocytes were lysed at room temperature in the dark for 10 minutes with a 1% lysis buffer (BioLegend, San Diego, CA, USA). Cells were washed by adding BD FACS Flow isotonic buffer, centrifuged at 500 G for 5 minutes, followed by decantation of the supernatant. The washing was repeated a total of three times. The isolated leukocytes were resuspended in isotonic buffer, stained with monoclonal fluorescent antibodies (see details in Supplementary Table S2), and incubated for 20 minutes at room temperature in the dark. The stained leukocytes were washed in isotonic buffer and analyzed on the BD FACS Canto II flow cytometer (BD Bioscience, San Jose, CA, USA) with a gating size of 100,000 cells. Flow cytometry data was analyzed using FlowJo (Tree Star, Ashland, OR, USA; version 10.10.0). Gating was performed with Boolean sequences by identifying singlet lymphocytes and monocytes. Lymphocytes were gated for CD4 and CD8 to identify CD4+ and CD8+ T cells. Monocytes were gated for CD14 and CD16 to identify classical (CD14^high^CD16^low^), intermediate (CD14^high^CD16^high^), and non-classical (CD14^low^CD16^high^) subtypes. T cells were analyzed for the expression levels of CCR1, CCR2, CCR5, CCR6, and CXCR3, as these chemokine receptors on T cells have been suggested to be implicated in AMD pathogenesis.^17^^–^^21^ Monocytes were analyzed for the expression levels of CCR1, CCR2, CCR5, CXCR2, CXCR3, and CX_3_CR1, as these chemokine receptors on monocytes have been suggested to be implicated in AMD pathogenesis.^15^^,^^16^^,^^22^^,^^23^ An example of the gating strategy can be found in Figure 1.

### Genotyping

A detailed description of the genotyping has been reported previously.^6^ In brief, genotyping was performed in patients with nAMD on full blood for the single nucleotide polymorphisms CFH rs1061170 and ARMS2 rs10490924. The blood was frozen at –80°C within 1 hour to be analyzed another day. Genotyping was performed by BioXpedia, Aarhus, Denmark, with the Fluidigm GT192.24 Dynamic Array Integrated Fluidic Circuit (Fluidigm Corp., San Francisco, CA, USA) according to the manufacturer’s guidelines.

### Statistics

Analysis of covariance (ANCOVA) was performed in group comparisons adjusted for age and smoking status (never or previous smoker). Histograms and the Shapiro-Wilk test were performed to evaluate the normality of the distributions. Logarithmic transformation was performed to normalized skewed data for ANCOVA. Healthy controls were chosen as reference group in the AMD stage comparisons (healthy controls, iAMD, and nAMD) using ANCOVA. Good treatment response was chosen as the reference group in nAMD treatment response comparisons (good, partial, and poor) using ANCOVA. Results of ANCOVA are presented as mean difference and 95% confidence interval (95% CI) in percentages. Visual acuity group comparisons were tested with analysis of variance (ANOVA) and reported as mean and standard deviation (SD). The chemokine-chemokine receptor interactions are visualized and described with correlation networks, with nodes representing the variables and the correlation as edges connecting them. Correlations were determined from Pearson's correlation coefficient with a threshold of |*r*| > 0.4 and *P* < 0.05. Association between genotypes and chemokines and chemokine receptors in patients with nAMD was analyzed with Welch two sample *t*-test. The CXCL8 concentration measurement was in 30.2% of cases below the lower limit of detection (LLOD) defined as 2.5 SD above the blank control adjusted for the dilution factor, with a value of approximately 135 pg/mL. Thus, CXCL8 was analyzed as a binary variable being over or under the LLOD. Correlations between CXCL8 and genotype were tested with Fisher's exact test. A *P* value < 0.05 was interpreted as statistically significant. Statistical analyses were performed with R software version 4.2.3 (R Foundation for Statistical Computing, Vienna, Austria).

## Results

### Study Population

In the DANEART study, 61 healthy controls, 34 patients with iAMD, and 100 patients with nAMD were included, of which 94 patients with nAMD completed the 1-year follow-up. Among patients with nAMD, the initial response distribution was as follows: 61 (65%) were good responders, 26 (28%) were partial responders, and 7 (7%) were poor responders. The 1-year response distribution was 50 (53%) good, 33 (36%) partial, and 11 (11%) poor responders. Mean (SD) number of injections after 1 year was 6.06 (SD = 1.78). Visual acuity between treatment response groups did not differ significantly at any timepoint. Mean (SD) baseline BCVA of good, partial, and poor responders was 59.7 (SD = 15.7), 62.6 (SD = 14.3), and 57.7 (SD = 12.1) Early Treatment Diabetic Retinopathy Study (ETDRS) letters, respectively. Mean (SD) post-loading dose BCVA for good, partial, and poor responders was 64.2 (SD = 14.7), 65.3 (SD = 15.6), and 51.8 (SD = 15.0), respectively. Mean (SD) 1-year BCVA for good, partial, and poor responders was 66.75 (SD = 11.9), 65.2 (SD = 11.9), and 64.8 (SD = 17.5), respectively (*P* > 0.05 for all group comparisons). The full details of the characteristics of these participants have been described previously.^6^

### Chemokine System and Diagnosis

**Figure 2.**
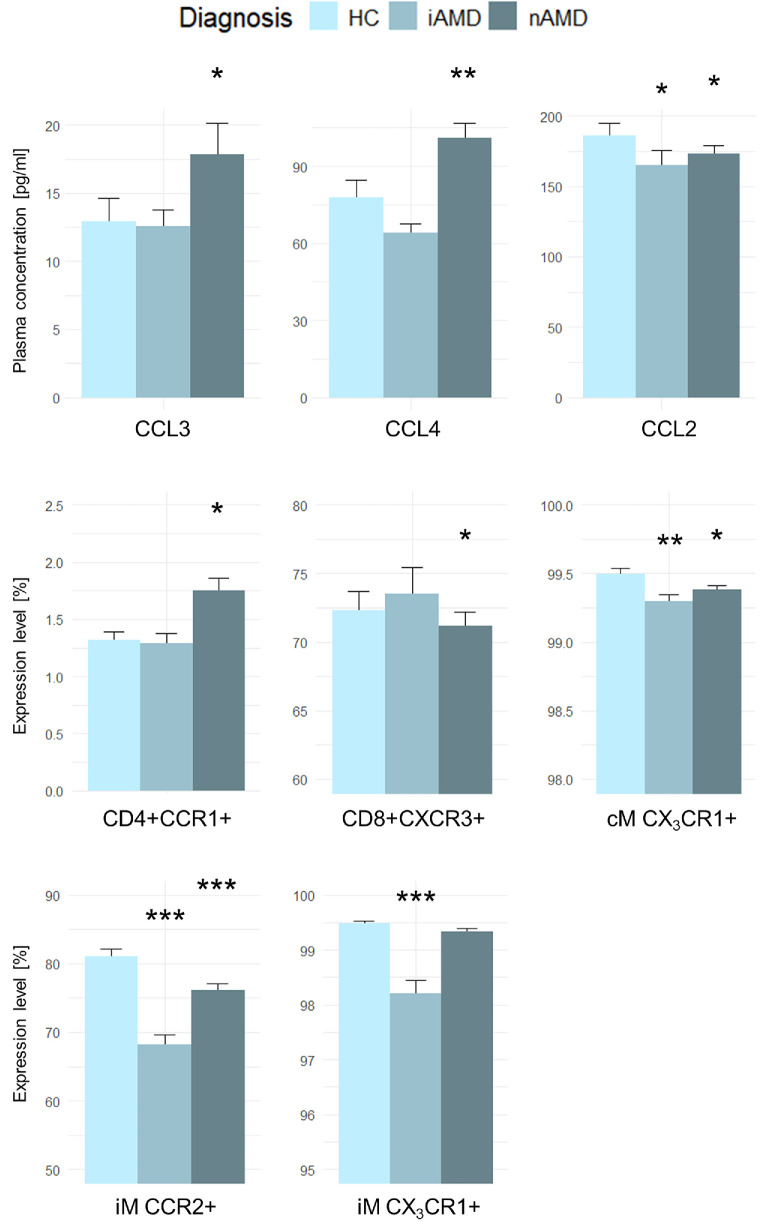
Chemokine and chemokine receptor levels significantly associated with diagnosis. HC, healthy controls; iAMD, intermediate age-related macular degeneration; nAMD, neovascular age-related macular degeneration; cM, classical monocytes; iM, intermediate monocytes. * *P* < 0.05; ** *P* < 0.01; *** *P* < 0.001 compared to the reference group (healthy controls) adjusted for age and smoking status.

Levels of the individual chemokines and chemokine receptors differed significantly according to diagnosis. CCL3 (mean difference = 20.8%, 95% CI = 2.4% to 39.2%, *P* = 0.027), CCL4 (mean difference = 26.8%, 95% CI = 9.6% to 44.1%, *P* = 0.002), and CD4+CCR1+ (mean difference = 20.7%, 95% CI = 3.2% to 38.1%, *P* = 0.021) were significantly elevated in patients with nAMD compared with healthy controls. CCL2 (mean difference = −13.5%, 95% CI = −24.3% to −2.5%, *P* = 0.016), CD8+CXCR3+ (mean difference = −2.9%, 95% CI = −5.7% to −0.1%, *P* = 0.041), CX_3_CR1+ classical monocytes (mean difference = −0.001%, 95% CI = −0.003% to −0.000%, *P* = 0.015), and CCR2+ intermediate monocytes (mean difference = −24.2%, 95% CI = −38.5 to −10.1, *P* < 0.001) were significantly decreased in patients with nAMD compared with healthy controls. CCL2 (mean difference = −16.2%, 95% CI = −31.0 to −1.3%, *P* = 0.032), CX_3_CR1+ classical monocytes (mean difference = −0.002%, 95% CI = −0.003% to −0.001%, *P* = 0.004), CCR2+ intermediate monocytes (mean difference = −59%, 95% CI = −76.2% to −42.1%, *P* < 0.001), and CX_3_CR1+ intermediate monocytes (mean difference = −1.2%, 95% CI = −0.8% to −1.5%, *P* < 0.001) were significantly decreased in patients with iAMD compared with healthy controls (Fig. 2). All results of the correlations between the chemokine system and diagnosis can be found in Supplementary Figure S1.

### Chemokine System and Treatment Response of Patients With nAMD 

**Figure 3.**
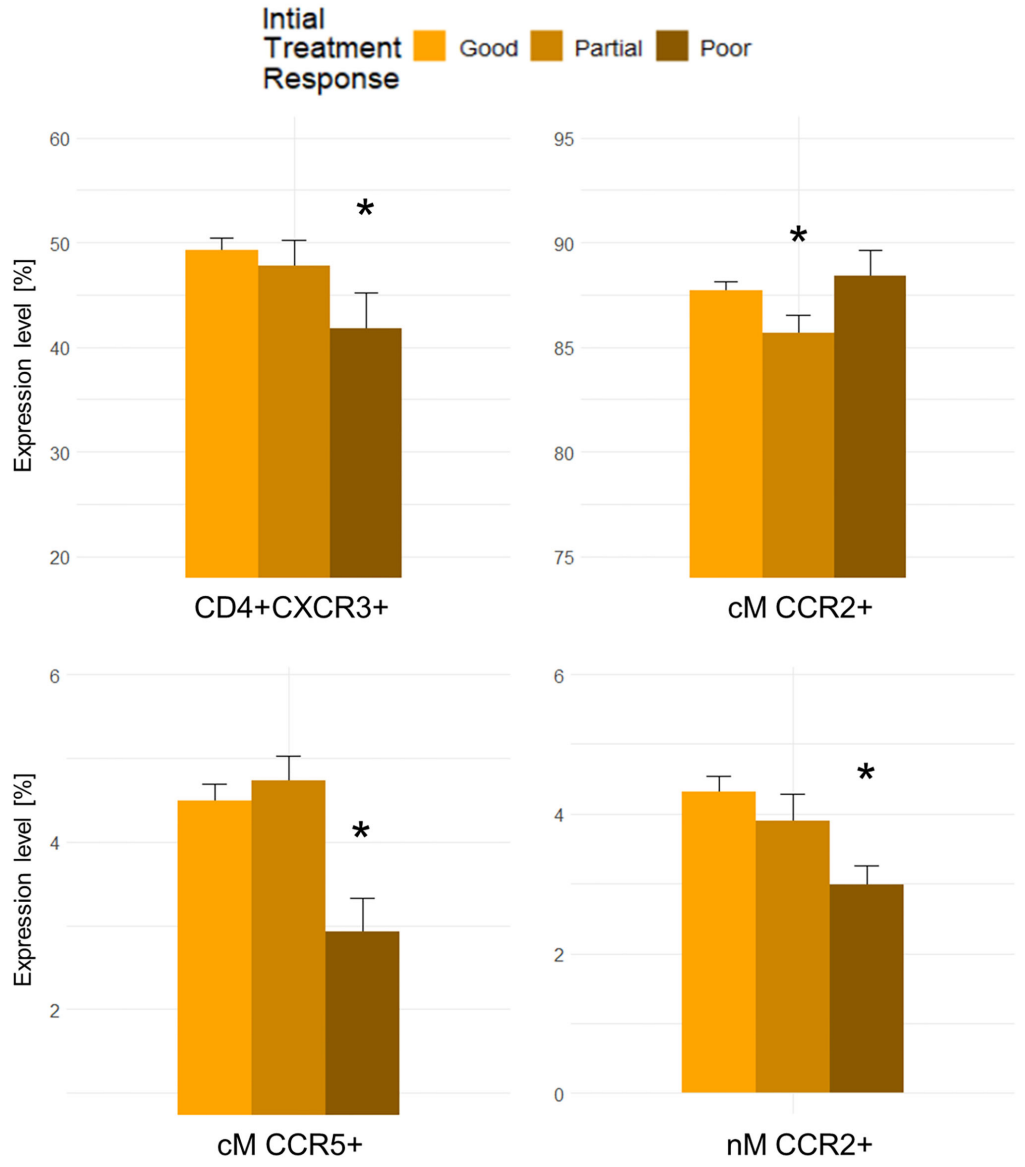
Chemokine receptor levels significantly associated with initial treatment response. cM, classical monocytes; nM, non-classical monocytes. * *P* < 0.05 compared to the reference group (good responders) adjusted for age and smoking status.

**Figure 4.**
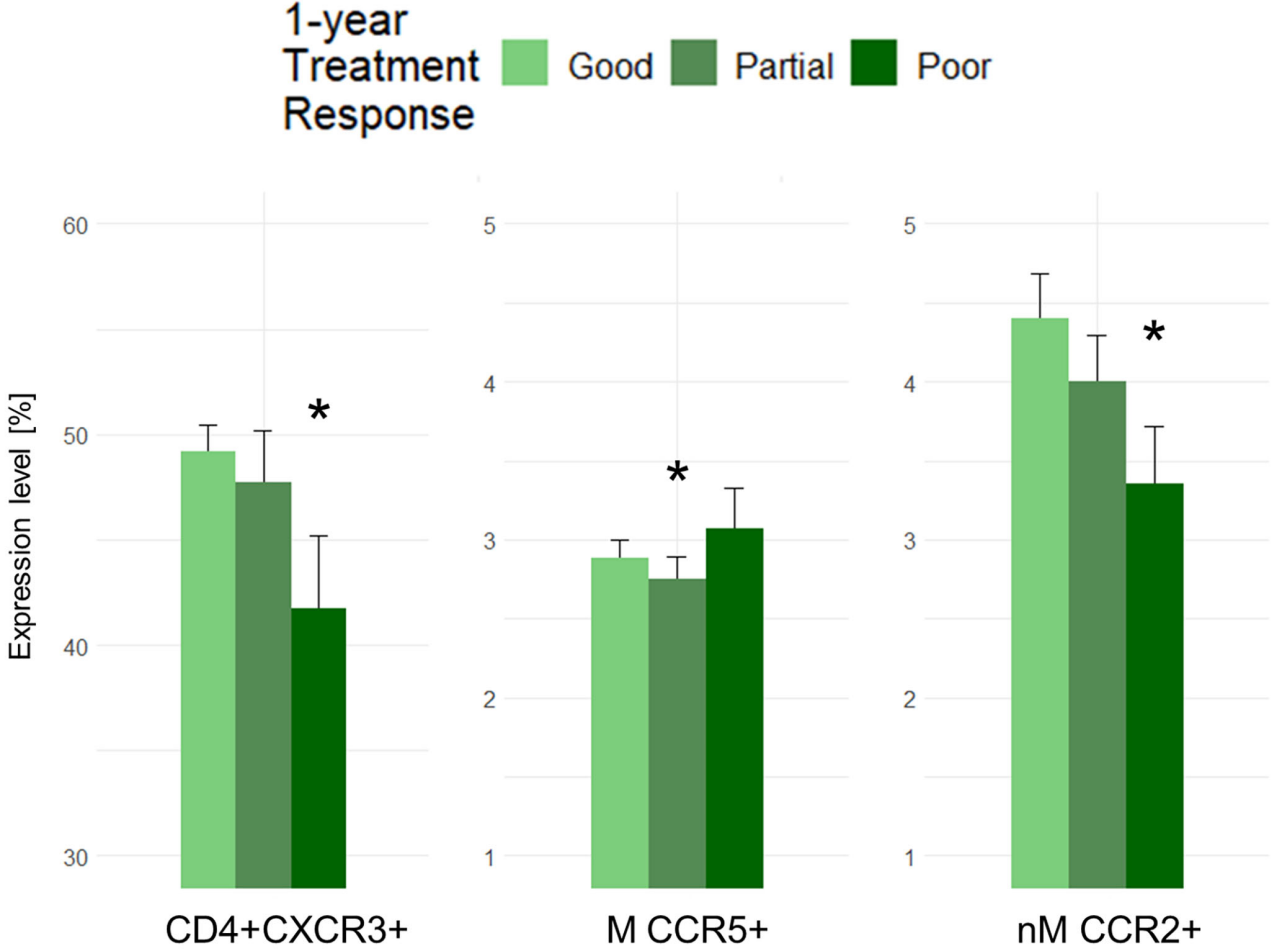
Chemokine receptor levels significantly associated with 1-year treatment response. M, monocytes; nM, non-classical monocytes. * *P* < 0.05 compared to the reference group (good responders) adjusted for age and smoking status.

Levels of chemokine system components were compared across nAMD treatment response groups (good, partial, and poor) with good responders as the control group. Poor initial treatment response in patients with nAMD had significantly decreased proportions of CD4+CXCR3+ (mean difference = –9.2%, 95% CI = –18.1% to –0.2%, *P* = 0.046), CCR5+ classical monocytes (mean difference = –36.2%, 95% CI = –67.1% to –4.4%, *P* = 0.029), and CCR2+ non-classical monocytes (mean difference = –38.8%, 95% CI = –73.9% to –4.2%, *P* = 0.031) compared with good initial responders. Partial initial treatment response was significantly associated with lower proportions of CCR2+ classical monocytes (mean difference = –1.8%, 95% CI = –2.5% to –0.1%, *P* = 0.044) compared with good initial responders (Fig. 3). There was a tendency of increased CXCL10 in poor initial responders compared with good initial responders (mean difference = 41.2%, 95% CI = –4.5% to 86.8%, *P* = 0.076). Poor 1-year treatment response was significantly associated with CD4+CXCR3+ (mean difference = –9.7, 95% CI = –18.0% to –1.2%, *P* = 0.025), and CCR2+ non-classical monocytes (mean difference = –34.3%, 95% CI = –66.9% to –1.4%, *P* = 0.042) compared with good 1-year responders. Partial 1-year treatment response was significantly associated with CCR5+ monocytes (mean difference = –15.2%, 95% CI = –27.5% to –2.2%, *P* = 0.026) compared to good 1-year response (Fig. 4). There was a tendency of increased CXCL10 in poor 1-year responders compared to good 1-year responders (mean difference = 35.2%, 95% CI = –4.6% to 75.1%, *P* = 0.082). All results of the correlations between the chemokine system and treatment response can be found in Supplementary Figures S2 and S3.

### Correlation Networks

**Figure 5.**
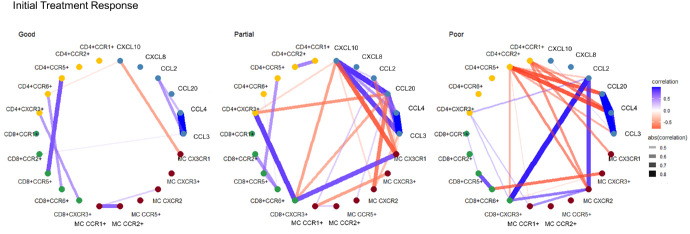
Correlation networks of the chemokine system according to initial treatment response in patients with neovascular age-related macular degeneration. Chemokines and chemokine receptors (nodes) are connected by correlations (edges), with a threshold of |*r*| > 0.4 and *P* < 0.05. MC, monocytes.

**Figure 6.**
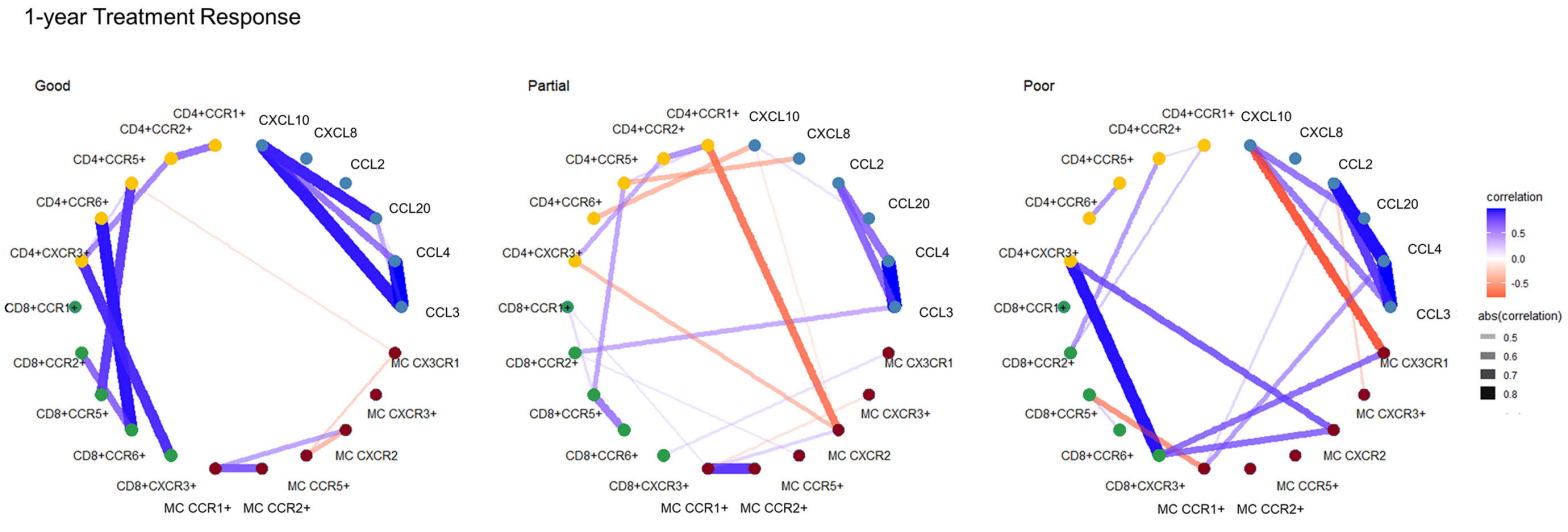
Correlation networks of the chemokine system according to 1-year treatment response in patients with neovascular age-related macular degeneration. Chemokines and chemokine receptors (nodes) are connected by correlations (edges), with a threshold of |*r*| > 0.4 and *P* < 0.05. MC, monocytes.

To describe the interactions among chemokines and chemokine receptors on CD4+ T cells, CD8+ T cells, and monocytes, we performed a correlation network analysis for each treatment response group. The link between analytes can highlight interactions, and centrality can describe the importance of the analytes in the groups. Correlation networks between initial treatment response seemed to differ between the groups. Partial and poor initial responders appeared to have more complex chemokine correlation networks compared to good responders (26, 25, and 10 connections, respectively), reflecting the increased regulation (Fig. 5). The 1-year treatment response groups did not seem to differ greatly in complexity, but unique correlations were found in each group (Fig. 6). The correlation networks between good initial and good 1-year responders appear similar, whereas those of partial and poor responders differ notably. These findings suggest that chemokine correlation networks may be more effective in predicting good responses compared with partial or poor responses. The positive correlations among CCL3, CCL4, and CCL20 were consistent in all treatment response groups both initially and after 1 year. These might thus add negligible information regarding prediction of treatment response.

### Single Nucleotide Polymorphisms

There was a significant association between the high-risk CFH rs1061170 genotypes (CC or CT) and elevated levels of CCL4 (*P* = 0.028), CD4+CCR2+ T cells (*P* = 0.002), CD8+CCR2+ T cells (*P* = 0.002), and CXCR2+ intermediate monocytes (*P* = 0.049; Table 3). The high-risk ARMS2 rs10490924 genotypes (TT/TG) was also significantly associated with elevated expression levels of CX_3_CR1+ monocytes and classical monocytes (*P* = 0.013 and *P* = 0.010, respectively), CCR1+ non-classical monocytes (*P* = 0.036, respectively), as well as decreased levels of CCR1+ intermediate monocytes (*P* = 0.049), although these differences were quite minor (Table 4).

**Table 3.** Chemokines and Chemokine Receptor Levels Stratified According to CFH, rs1061170 Genotype

	CFH, rs1061170		
	CC/CT (High Risk), *n* = 82	TT (Low Risk), *n* = 18	*P* Value
Plasma Chemokines, Mean (SD)			
CCL3, pg/mL	18.35 (1.70)	15.45 (1.44)	0.96
CCL4, pg/mL	105.78 (1.58)	79.18 (1.40)	**0.028**
CCL20, pg/mL	15.57 (1.96)	15.42 (2.80)	0.46
CCL2, pg/mL	175.03 (1.34)	165.31 (1.28)	0.64
CXCL10, pg/mL	1379.69 (1.76)	1198.63 (1.65)	0.41
CXCL8, % above lower detection limit	66.67%	81.25%	0.37^*^
CD4+ T cells, %, mean (SD)			
CCR1+	1.78 (1.71)	1.60 (1.59)	0.56
CCR2+	1.27 (1.25)	0.80 (0.80)	**0.041**
CCR5+	2.89 (2.16)	3.13 (1.77)	0.28
CCR6+	26.14 (10.02)	26.49 (8.00)	0.88
CXCR3+	48.49 (11.12)	46.92 (8.95)	0.55
CD8+ T cells %, mean (SD)			
CCR1+	24.90 (1.54)	27.59 (1.75)	0.73
CCR2+	3.50 (1.79)	1.53 (2.10)	**0.002**
CCR5+	17.43 (2.65)	18.92 (3.48)	0.73
CCR6+	9.87 (2.94)	5.23 (1.62)	0.72
CXCR3+	71.18 (10.63)	71.24 (8.31)	0.98
Monocytes %, mean (SD)			
CCR1+	97.25 (1.01)	97.21 (1.01)	0.46
CCR2+	78.82 (4.55)	77.92 (3.53)	0.39
CCR5+	2.84 (1.31)	3.03 (1.45)	0.71
CXCR2+	84.40 (1.12)	83.24 (1.14)	0.97
CXCR3+	64.14 (4.70)	65.02 (4.80)	0.51
CX_3_CR1+	98.52 (1.02)	98.85 (1.01)	0.23
Classical monocytes %, mean (SD)			
CCR1+	97.25 (1.01)	97.21 (1.01)	0.46
CCR2+	87.05 (3.83)	87.64 (3.43)	0.54
CCR5+	95.55 (1.02)	95.45 (1.02)	0.73
CXCR2+	88.04 (3.25)	87.94 (4.93)	0.94
CXCR3+	63.62 (5.24)	64.61 (5.69)	0.53
CX_3_CR1+	99.37 (0.30)	99.46 (0.28)	0.27
Intermediate monocytes %, mean (SD)			
CCR1+	86.83 (1.07)	86.59 (1.06)	0.79
CCR2+	76.44 (1.15)	74.39 (1.12)	0.23
CCR5+	8.53 (1.76)	8.38 (1.77)	0.94
CXCR2+	84.44 (1.08)	81.51 (1.07)	**0.049**
CXCR3+	65.47 (7.01)	64.38 (4.33)	0.42
CX_3_CR1+	99.32 (0.60)	99.38 (0.45)	0.76
Non-classical monocytes %, mean (SD)			
CCR1+	48.45 (9.98)	48.61 (9.55)	0.95
CCR2+	4.08 (1.54)	4.22 (1.43)	0.53
CCR5+	8.12 (1.61)	6.29 (1.61)	0.068
CXCR2+	8.90 (2.00)	7.34 (3.32)	0.30
CXCR3+	68.43 (1.10)	68.13 (1.06)	0.51
CX_3_CR1+	90.59 (1.09)	93.31 (1.06)	0.12

Bold values indicate statistical significance (*P* < 0.05).

Statistical test: Welch two sample *t*-test.

* Fisher's exact test.

**Table 4.** Chemokines and Chemokine Receptor Levels Stratified According to ARMS2, rs10490924 Genotype

	ARMS2, rs10490924		
	TT/TG (High Risk), *n* = 45	GG (Low Risk), *n* = 55	*P* Value
Plasma Chemokines, Mean (SD)			
CCL3, pg/mL	14.75 (1.88)	21.79 (1.45)	0.20
CCL4, pg/mL	99.78 (1.64)	102.80 (1.51)	0.88
CCL20, pg/mL	17.15 (1.99)	13.53 (2.20)	0.30
CCL2, pg/mL	172.11 (1.31)	174.86 (1.39)	0.66
CXCL10, pg/mL	1300.27 (1.80)	1408.53 (1.69)	0.50
CXCL8, % above lower detection limit	70.00	68.63	0.99^*^
CD4+ T cells %, mean (SD)			
CCR1+	1.66 (0.77)	1.86 (1.40)	0.76
CCR2+	1.28 (1.66)	1.08 (0.51)	0.61
CCR5+	2.86 (1.70)	3.02 (2.56)	0.35
CCR6+	27.16 (8.97)	24.98 (10.50)	0.28
CXCR3+	47.86 (9.96)	48.69 (11.80)	0.71
CD8+ T cells %, mean (SD)			
CCR1+	25.29 (1.46)	25.41 (1.66)	0.61
CCR2+	3.35 (1.90)	3.17 (1.65)	0.99
CCR5+	18.87 (2.18)	16.18 (3.46)	0.14
CCR6+	9.97 (2.12)	8.01 (1.64)	0.26
CXCR3+	71.13 (10.57)	71.27 (9.95)	0.95
Monocytes %, mean (SD)			
CCR1+	97.28 (1.02)	97.19 (1.01)	0.94
CCR2+	78.55 (3.98)	78.76 (4.73)	0.81
CCR5+	2.77 (1.37)	3.01 (1.31)	0.24
CXCR3+	63.91 (4.16)	64.75 (5.32)	0.40
CXCR2+	84.70 (1.09)	83.57 (1.15)	0.80
CX_3_CR1+	98.81 (1.00)	98.27 (1.02)	**0.013**
Classical monocytes %, mean (SD)			
CCR1+	97.29 (1.01)	97.19 (1.02)	0.94
CCR2+	87.07 (3.88)	87.25 (3.64)	0.82
CCR5+	95.44 (1.02)	98.44 (1.02)	0.66
CXCR3+	63.41 (4.57)	64.26 (6.12)	0.45
CXCR2+	88.06 (3.38)	87.98 (3.81)	0.91
CX_3_CR1+	99.45 (0.30)	99.30 (0.27)	**0.010**
Intermediate monocytes %, mean (SD)			
CCR1+	85.64 (1.05)	88.24 (1.07)	**0.049**
CCR2+	76.46 (1.15)	75.66 (1.13)	0.63
CCR5+	8.14 (1.75)	8.97 (1.77)	0.39
CXCR3+	64.73 (6.33)	65.99 (7.01)	0.36
CXCR2+	83.44 (1.08)	84.60 (1.07)	0.42
CX_3_CR1+	99.34 (0.64)	99.32 (0.49)	0.34
Non-classical monocytes %, mean (SD)			
CCR1+	50.33 (9.81)	46.14 (9.54)	**0.036**
CCR2+	4.20 (1.47)	3.99 (1.58)	0.39
CCR5+	7.68 (1.62)	7.96 (1.63)	0.83
CXCR3+	67.75 (6.34)	69.16 (5.86)	0.29
CXCR2+	8.42 (2.10)	8.90 (2.01)	0.43
CX_3_CR1+	91.33 (1.09)	90.67 (1.08)	0.64

Bold values indicate statistical significance (*P* < 0.05).

Statistical test: Welch two sample *t*-test.

* Fisher's exact test.

## Discussion

Angiogenesis is a crucial factor in nAMD pathogenesis and treatment. The MNV formation in these patients is highly mediated by stimulation from VEGF and current treatment aims to inhibit this pro-angiogenic growth factor. The chemokine system has important angiogenic properties and has previously been shown to be affected in patients with AMD.^5^ The chemokine system is a highly complex and integrated system.^31^ Chemokine receptors are dynamic and adapt to environmental stimuli, with ligand bias and allosteric regulation causing activation of multiple pathways.^32^ Additionally, the system modulates immune responses and T cell differentiation, influencing inflammation, angiogenesis, wound healing, and scar formation.^33^^–^^35^ Previous studies have found increased levels of systemic pro-angiogenic chemokines and receptors in patients with nAMD. However, these studies have investigated few ligand-receptor axes,^22^ chemokines in isolation^12^^,^^14^ or chemokine receptors in isolation.^15^^–^^17^^,^^20^^,^^21^^,^^23^ In the same regard, the association between the chemokine system and treatment response in patients with nAMD is sparsely investigated,^22^^,^^23^ as well as in patients with iAMD.^13^^,^^20^^,^^22^

In this study, we find plasma concentrations of CCL3 and CCL4 to be significantly increased in patients with nAMD compared with healthy controls. Zhou et al. did not find a significant difference of CCL3 between patients with nAMD and healthy controls, but did observe a significant difference for CCL4.^12^ Activated monocytes and T cells can secrete CCL3 and CCL4, which can recruit monocytes and T cells,^36^ and has been shown to be increased with aging.^37^ We find increased levels of circulating CD4+CCR1+ T cells in patients with nAMD compared with healthy controls. CCL3 can activate CD4+CCR1+ T cells, which will be recruited to sites of inflammation and promote their pro-inflammatory properties.^8^ This includes inducing a pro-angiogenic environment, which may contribute to the neovascularization present in nAMD. The proportion of the anti-inflammatory CD8+CXCR3+ T cells was decreased in patients with nAMD, which causes a dysregulation of inflammation and angiogenesis, which has also been observed previously by Falk et al.^20^ We also find decreased levels of plasma CCL2 and CCR2+ intermediate monocytes in patients with iAMD and patients with nAMD compared with healthy controls. The CCL2-CCR2 axis is involved in the chemotaxis and inflammation of monocytes. The downregulation of this axis suggests that there might be a decreased activation of circulating monocytes systemically.^38^ Similarly CX_3_CR1+ classical monocytes were statistically significantly decreased in patients with iAMD and patients with nAMD, as well as CX_3_CR1+ intermediate monocytes in patients with iAMD, suggesting these cells might be especially involved in iAMD pathogenesis.

Patient treatment response varies and patients with poor and partial response need improved treatment options. Because of the previously found alterations of the chemokine system and the difference found in this study between patients with nAMD and healthy controls, we investigated whether differences of specific chemokines and chemokine receptors are associated with treatment response. The patients with nAMD with poor initial and 1-year treatment response had lower proportions of CD4+CXCR3+ T cells. There was also a tendency of poor initial treatment responders to have elevated levels of the ligand CXCL10. The lower proportions of the anti-angiogenic circulating CD4+CXCR3+ T cells and tendency of increased CXCL10 might cause angiogenesis. As CXCL10 binds to CXCR3, leading to the internalization of the receptor,^39^ this observation is consistent with the expected outcome. Dysregulation and blocking of CXCR3 has been shown to cause angiogenesis, and the CXCL10-CXCR3 axis mediates VEGF-induced angiogenesis.^40^ Thus, treatment with anti-VEGF injections might not be sufficient to dampen the pro-angiogenic environment of the retina in poor responders. We have previously reported that interferon-γ is elevated in poor treatment responders, which induces the production of CXCL10.^41^

Proportions of the pro-angiogenic CCR5+ classical and CCR2+ non-classical monocytes were lower in poor initial responders, as well as CCR2+ monocytes in partial responders. Similarly, proportions were decreased of CCR2+ non-classical monocytes in poor 1-year responders, as well as CCR5+ monocytes in partial 1-year responders. The lower proportions of CCR2 and CCR5 might contribute to the development of nAMD by disrupting the balance of inflammation resolution and angiogenesis in the retina. CCR2 and CCR5 recruit monocytes, where they differentiate into macrophages that resolve inflammation and regulate angiogenesis.^42^ A reduction of CCR2 in the poor and partial responders might lead to insufficient macrophage-mediated clearance causing chronic inflammation and angiogenesis in the retina. This would lead to increased VEGF production, and the conventional anti-VEGF injections might not be potent enough to reduce the leakage and regression of the MNV.

Most of the statistically significant differences found in this study are minor and might have limited biological significance. As AMD is characterized by chronic low-grade inflammation, it was expected that individual chemokines would be only slightly affected. Thus, correlation networks were generated, which can reveal immune regulation dynamics in complex integrated systems. The correlation networks showed substantial differences between the groups. Initial treatment response groups of patients with nAMD showed different correlation phenotypes, where partial and poor initial responders seemed to have a more complex correlation network compared with good responders. The complexity indicates a more pro-inflammatory regulation in these groups, which could cause the dysregulation of angiogenesis systemically. A definitive pattern after 1 year was less apparent, and the chemokine correlation profile might thus be of most value post-loading dose. This was expected, as patients will be subjected to differing treatment intervals and confounding factors after 1 year, rather than the similar initial treatment course.

The 2 main risk polymorphisms for developing AMD, CFH rs1061170 and ARMS2 rs10490924, are also important in activation of the complement system.^24^ In this study, we evaluated the effect of these polymorphisms on the chemokine system in patients with nAMD. We found the CFH risk genotypes were associated with elevated levels of CCL4, CD4+CCR2+ T cells, CD8+CCR2+ T cells, and CXCR2+ intermediate monocytes. The ARMS2 risk genotype was associated with CX_3_CR1+ monocytes and classical monocytes, as well as CCR1+ intermediate monocytes and non-classical monocytes. This adds to our understanding that genetic susceptibility is involved in the low-grade inflammation in nAMD, caused by the chemokine system, at least in part. The differences were statistically significant but were minor. However, genetic risk factors in chemokine receptor genes CX_3_CR1 and CCR3 has previously been identified in AMD, which supports the primary role of the chemokine system in AMD pathogenesis.^43^^,^^44^

Limitations of this study include the observational design, which is unable to determine whether these differences in the chemokine system are causal or reflect an adaptation to the condition. The categorical groupings build on previous classifications focused on clinical implementation^1^^,^^2^^,^^45^; however, this approach restricts the capture of subtle differences. The relatively small number of patients in the poor response group was also a limitation, as it may obscure potentially significant correlations. Because the analyses in this study build upon existing evidence of specific dysfunctional pathways in the chemokine system in nAMD, it might not be necessary to adjust for multiple testing.^46^ However, given the large number of statistical tests, further investigation is required to confirm the significance of the chemokine system in the treatment response of nAMD. Future studies should validate these findings in other study populations to robustly establish the described associations. Investigating changes in the chemokine system before and after treatment would enhance clinical relevance by providing insights into the dynamics of these pathways and their role in treatment response.

In conclusion, our findings underscore the critical role of the chemokine system in nAMD pathogenesis and treatment response. Dysregulation of chemokine-mediated inflammation and angiogenesis may contribute to disease progression and therapeutic resistance. Further studies are warranted to explore targeted therapies that modulate the chemokine system to improve outcomes in; nAMD, particularly those with poor or partial response to anti-VEGF treatment.

## Supplementary Material

Supplement 1

Supplement 2

Supplement 3
