# Supplementary material for: Chemokine System Changes Drive Age-Related Macular Degeneration and Influence Treatment Outcomes
Source: Invest Ophthalmol Vis Sci. 2025 May 6;66(5):14. doi: 10.1167/iovs.66.5.14 (PMC12061063; doi:10.1167/iovs.66.5.14)
Supplement: Supplement 1 [file iovs-66-5-14_s001.pdf]

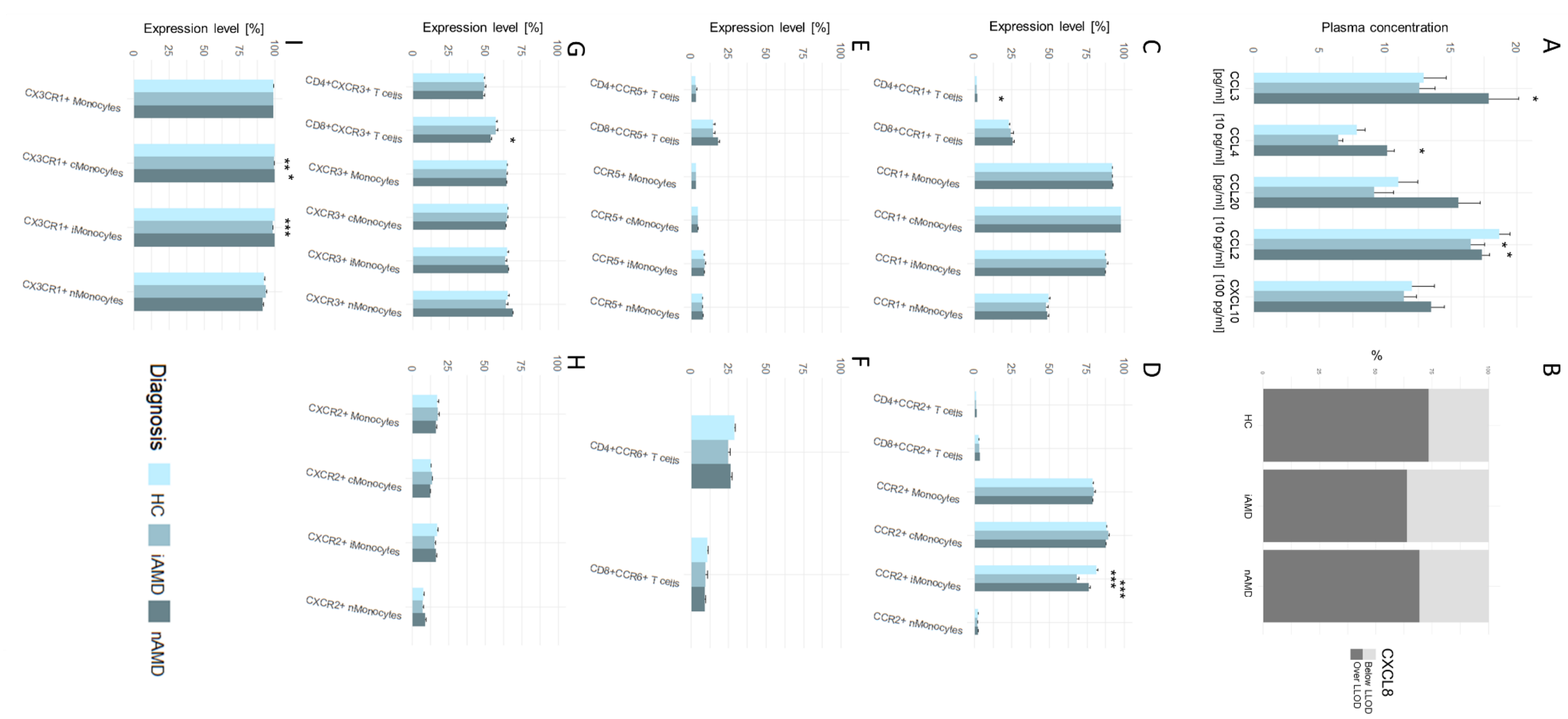

Supplementary figure S1*. Chemokine and chemokine receptor levels according to diagnosis. HC = healthy controls; iAMD = intermediate age-related macular degeneration; nAMD = neovascular age-related macular degeneration; LLOD = lower level of detection; cMonocytes = classical monocytes; iMonocytes = intermediate monocytes; nMonocytes = non-classical monocytes. * P < 0.05; ** P < 0.01; *** P < 0.001 compared to the reference group (healthy controls) adjusted for age and smoking status.*

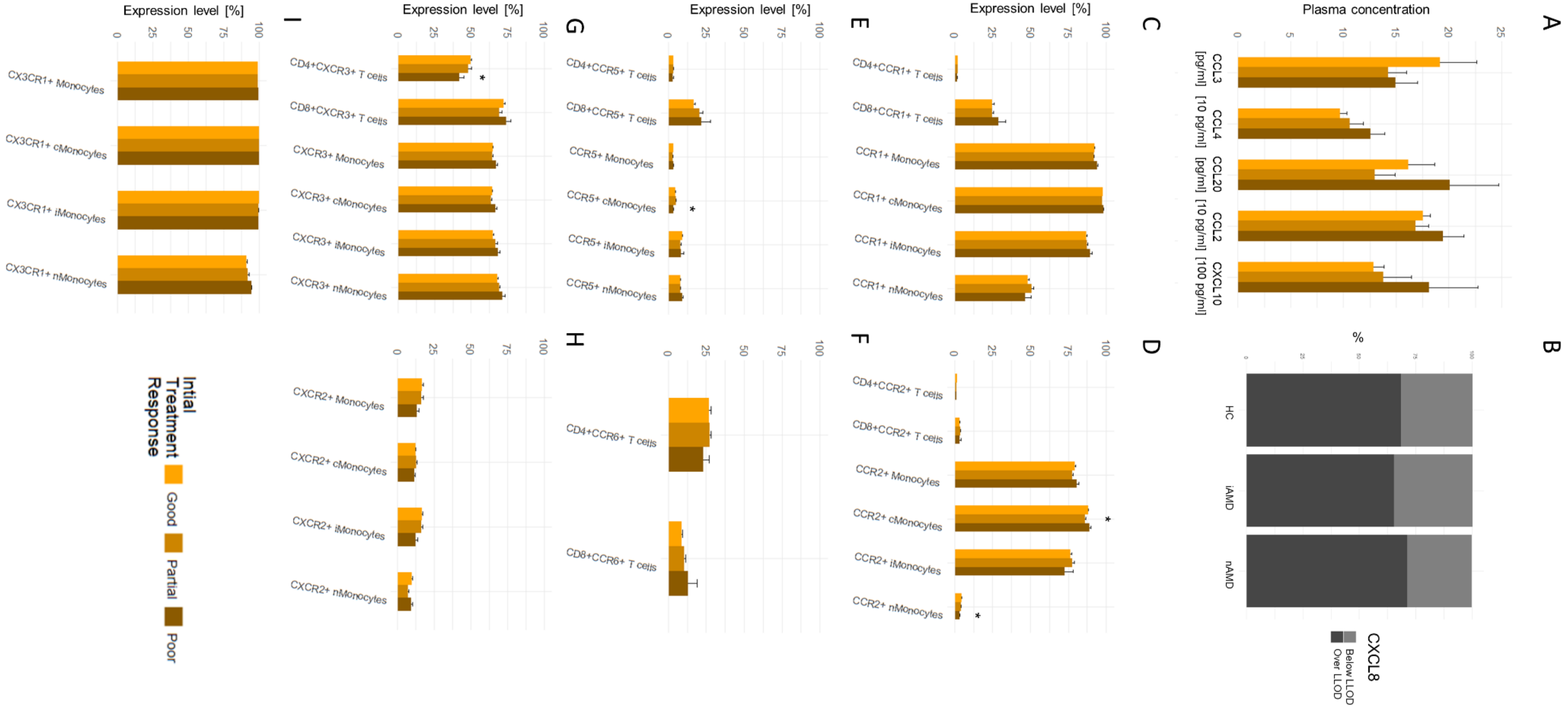

***Supplementary figure S2**. Chemokine and chemokine receptor levels according to initial treatment response. LLOD = lower level of detection; cMonocytes = classical monocytes; iMonocytes = intermediate monocytes; nMonocytes = non-classical monocytes. * P < 0.05 compared to the reference group (good responders) adjusted for age and smoking status.*

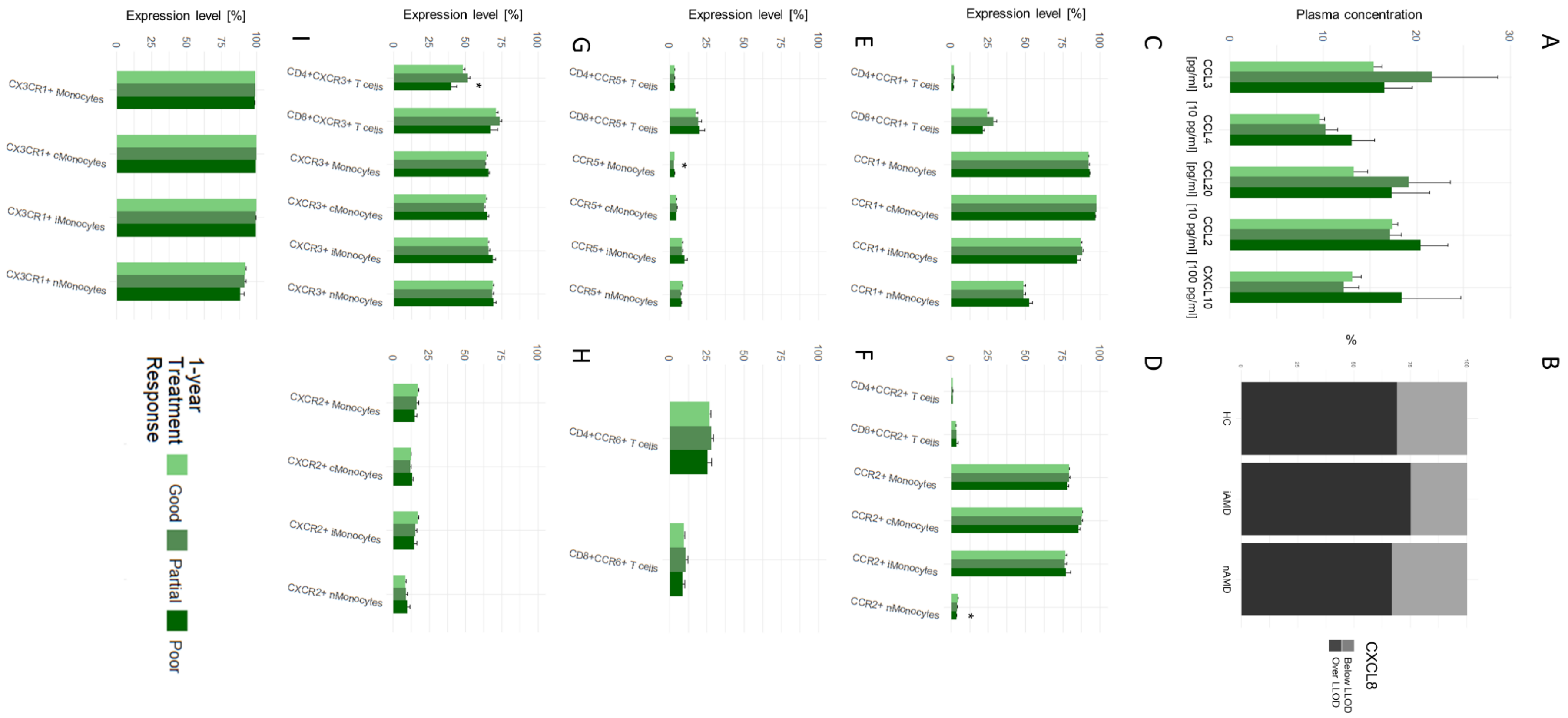

Supplementary figure S3. *Chemokine and chemokine receptor levels according to 1-year treatment response. LLOD = lower level of detection; cMonocytes = classical monocytes; iMonocytes = intermediate monocytes; nMonocytes = non-classical monocytes. * P < 0.05 compared to the reference group (good responders) adjusted for age and smoking status.*
