# Supplementary material for: Chemokine System Changes Drive Age-Related Macular Degeneration and Influence Treatment Outcomes
Source: Invest Ophthalmol Vis Sci. 2025 May 6;66(5):14. doi: 10.1167/iovs.66.5.14 (PMC12061063; doi:10.1167/iovs.66.5.14)
Supplement: Supplement 2 [file iovs-66-5-14_s002.pdf]

Supplementary table S1. Chemokine assays.

**Analytes**	**CCL2, CCL3, CCL4, CXCL8, CXCL10**	**CCL20**
**Cytokine assay**	V-PLEX Chemokine Panel 1 (human)	V-PLEX TH17 Panel 1 (human)
**Catalog number**	K151A9H-1	K15085D-1
Abbreviations: CCL = C-C motif chemokine ligand, CXCL = C-X-C motif chemokine ligand. Manufacturer: Mesoscale Discovery, Rockville, MD, USA.		
