# Supplementary material for: Chemokine System Changes Drive Age-Related Macular Degeneration and Influence Treatment Outcomes
Source: Invest Ophthalmol Vis Sci. 2025 May 6;66(5):14. doi: 10.1167/iovs.66.5.14 (PMC12061063; doi:10.1167/iovs.66.5.14)
Supplement: Supplement 3 [file iovs-66-5-14_s003.pdf]

Supplementary table S2. Flow cytometry antibodies.

Surface marker	Fluorochrome	Manufacturer	Catalog number
CD4	PerCP	R&D Systems	FAB3791C
CD8	Brilliant Violet	BioLegend	301048
CD14	Pacific Blue	BioLegend	325616
CD16	APC-Cy7	BioLegend	302018
CCR1	APC	BioLegend	362908
CCR2	PE	R&D Systems	FAB151P
CCR5	FITC	R&D Systems	FAB182F
CCR6	FITC	BioLegend	353412
CXCR2	APC	BioLegend	320710
CXCR3	PE/Cy7	BioLegend	353720
CX_3CR1	FITC	BioLegend	341606

APC= Allophycyanin, APC/Cy7 = Allophycocyanin-cyanine 7, FITC = Fluorescein isothiocyanate, PE = Phycoerythrin, PE/Cy7 = Phycoerythrin-cyanine 7, PerCP = Peridinin-chlorophyll-protein.

R&D Systems, Minneapolis, MN, USA.

BioLegend, San Diego, CA, USA.
